# Establishing the role of honest broker: bridging the gap between protecting personal health data and clinical research efficiency

**DOI:** 10.7717/peerj.1506

**Published:** 2015-12-17

**Authors:** Hyo Joung Choi, Min Joung Lee, Chang-Min Choi, JaeHo Lee, Soo-Yong Shin, Yungman Lyu, Yu Rang Park, Soyoung Yoo

**Affiliations:** 1 Office of Clinical Research Information, Asan Institute of Life Sciences, Asan Medical Center, Seoul, Korea; 2 Department of Pulmonology and Critical Medicine, Asan Medical Center, University of Ulsan College of Medicine, Seoul, Korea; 3 Department of Oncology, Asan Medical Center, University of Ulsan College of Medicine, Seoul, Korea; 4 Department of Biomedical Informatics, Asan Medical Center, Seoul, Korea; 5 Department of Emergency Medicine, Asan Medical Center, University of Ulsan College of Medicine, Seoul, Korea; 6 Clinical Research Center, Asan Institute of Life Sciences, Asan Medical Center, Seoul, Korea; 7 Human Research Protection Center, Asan Institute of Life Sciences, Asan Medical Center, Seoul, Korea

**Keywords:** Honest broker, Research efficiency, Secondary use of clinical data, Patient health information protection

## Abstract

**Background.** The objective of this study is to propose the four conditions for the roles of honest brokers through a review of literature published by ten institutions that are successfully utilizing honest brokers. Furthermore, the study aims to examine whether the Asan Medical Center’s (AMC) honest brokers satisfy the four conditions, and examine the need to enhance their roles.

**Methods.** We analyzed the roles, tasks, and types of honest brokers at 10 organizations by reviewing the literature. We also established a Task Force (TF) in our institution for setting the roles and processes of the honest broker system and the honest brokers. The findings of the literature search were compared with the existing systems at AMC—which introduced the honest broker system for the first time in Korea.

**Results.** Only one organization employed an honest broker for validating anonymized clinical data and monitoring the anonymity verifications of the honest broker system. Six organizations complied with HIPAA privacy regulations, while four organizations did not disclose compliance. By comparing functions with those of the AMC, the following four main characteristics of honest brokers were determined: (1) de-identification of clinical data; (2) independence; (3) checking that the data are used only for purposes approved by the IRB; and (4) provision of de-identified data to researchers. These roles were then compared with those of honest brokers at the AMC.

**Discussion.** First, guidelines that regulate the definitions, purposes, roles, and requirements for honest brokers are needed, since there are no currently existing regulations. Second, Korean clinical research institutions and national regulatory departments need to reach a consensus on a Korean version of Limited Data Sets (LDS), since there are no lists that describe the use of personal identification information. Lastly, satisfaction surveys on honest brokers by researchers are necessary to improve the quality of honest brokers.

## Introduction

Prior to the age of big data, privacy was protected by individuals who maintained control over their own personal information. In other words, research subjects provided informed consent about (1) to whom, (2) how, (3) for what purpose, and (4) to what extent they would provide their personal information (Mayer-Schönberger & Cukier, 2013). However, in the current age of big data, “informed consent” may no longer be a realistic and efficient method for protecting privacy (Solove, 2012; Tene & Polonetsky, 2014). This is because there is an immense need for data sharing and interlinking of data for secondary research purposes, and this secondary use, in most cases, has not been planned when the data were originally collected and stored. It is also unrealistic to re-obtain consent from those who initially agreed to collection of their data (Mayer-Schönberger & Cukier, 2013).

Secondary use and informed consent are *not* exemptions in the area of clinical research. There has also been an increase in the number of studies that use clinical data that were previously collected by hospital information systems, such as electronic health records (EHRs). In practice, secondary uses of health data can enhance individual healthcare experiences, expand knowledge about diseases and treatments, strengthen healthcare system efficiency and effectiveness, and help businesses meet customer needs (Safran et al., 2007). However, especially in cases of retrospective secondary studies, it is often highly impractical or simply impossible to recontact all research subjects for new uses of their data. Furthermore, even if the researchers obtain consent from the subjects who are not representative of the original population, the study may turn out to be statistically skewed or underpowered (El Emam, Jonker & Fineberg, 2011; Kho et al., 2009; Macleod & Watt, 2008; Willison et al., 2008). These issues are not simply about obtaining consent from all subjects for secondary use or discarding all clinical data for which permission has not been given for secondary use. To resolve these problems, obtaining “broad” (i.e., “open” or “blanket”) consent for using data or biomaterials for purposes that cannot be initially foreseen has been proposed (Hofmann, 2009; Petrini, 2010). In comparison to tightly focused consent for studies on particular illnesses or medical techniques, broad consent is considered essential if the data in the EHR are linked to registries or human biomaterials data, or if there are other data that meet optimal research merits (Kosseim & Brady, 2008). However, this approach is also limited in that we can only use data or human biomaterials that were obtained following consent for broad purposes.

To address these challenges, many jurisdictions allow institutional review boards (IRBs) to waive obtainment of informed consent. The accepted criteria for a waiver in the US need to satisfy the stipulations of federal regulation 45 CFR46.116(d) (US Department of Health & Human Services, 2015): (1) the research involves no more than minimal risk to the subjects; (2) the waiver or alteration will not adversely affect the rights and welfare of the subjects; (3) the research could not practicably be carried out without the waiver or alteration; and, (4) whenever appropriate, the subjects will be provided with additional pertinent information after participation. Similarly, the Korean Bioethics and Safety Act (which was entirely amended in 2013) emphasizes that informed consent must be obtained from the participants in all human subject studies and human biological materials studies, including consent waiver clauses that explain the conditions for waiving informed consent requirements. IRBs may approve a researcher’s request to exempt the requirements to obtain informed consent if the following criteria are met: (1) it is realistically impossible for the researcher(s) to obtain informed consent from the research participants, or it is determined that consent could severely impact the feasibility of the research, and (2) there is no reason to assume that the research participants would deny consent and the risk of the research for the participants is extremely low.

One method that makes possible the secondary use of clinical data while also satisfying the aforementioned conditions for waiving consent is to intentionally anonymize direct and possible identifiers. In other words, although identifiers were available when the data were collected or stored, these identifiers can be removed or unlinked from the clinical data for the intentional purpose of satisfying the consent waiver and protecting participant information before sharing data. This international anonymization method meets a minimum threshold for confidentiality that is expected by privacy laws and regulations (El Eman, Rodgers & Malin, 2015). Using this method, there is no legal responsibility to discard clinical data that has run out of primary uses.

Nonetheless, ethical and technical concerns remain even after satisfying legal conditions. This is because sharing patient data for secondary purposes is reliant on patient trust, so there are expectations that data will be adequately anonymized, even though anonymization cannot guarantee that the risk of re-identification drops to zero (El Eman, Rodgers & Malin, 2015). Therefore, problems remain with how to ensure that (1) the identifiers are actually de-identified, and (2) there are guarantees against researchers re-identifying clinical data. Accordingly, the concept of the “honest broker” was devised. The original meaning of the term honest broker is an impartial mediator in international, industrial, or other disputes (Oxford English Dictionary, 2015). The definition is more specific, however, in clinical research, where it is defined as a neutral intermediary (person or computer system) between researchers, the individual whose tissue and data were collected, and the healthcare provider who obtained the tissue and/or data and thereby has a responsibility to protect personal identifiable information (which must be separated from the original clinical data) (Dhir et al., 2008).

Curiously, despite the increase in using honest brokers as intermediaries between protecting personal health data and promoting clinical research efficiency (Boyd et al., 2009; Children’s Brain Tumor Tissue Consortium, 2015; Dennis et al., 2006; Dhir et al., 2008; Liu et al., 2009; McConnell et al., 2008; University of Chicago, 2015; University of Arkansas for Medical Sciences, 2015; University of Florida, 2015; Wade et al., 2014), the role, process, and requirements of honest brokers have not been examined in detail. The aim of the present study was to establish the roles of honest brokers and provide sufficient empirical evidence that supports the need for those roles. Accordingly, we first analyzed the roles, tasks, and types of honest brokers at 10 organizations by reviewing the literature. These common roles were compared to those at Asan Medical Center (AMC)—where the honest broker system was adopted for the first time in Korea—to verify if the AMC brokers truly satisfy the concept of honest brokers. The AMC is the largest medical center in South Korea, with approximately 2,700 inpatient beds and 10,000 outpatient visits per day. Since its establishment in 1989, the hospital information system (HIS) has been actively used to improve quality of care and clinical research, and to make the clinical workflow more efficient. To utilize these clinical data, about 1,800 clinical data extractions are requested by investigators each year. Lastly, we argue the need to expand the roles of the honest broker and discuss several unexplored aspects of this issue.

## Materials and Methods

### Existing literature on honest brokers

**Figure 1:**
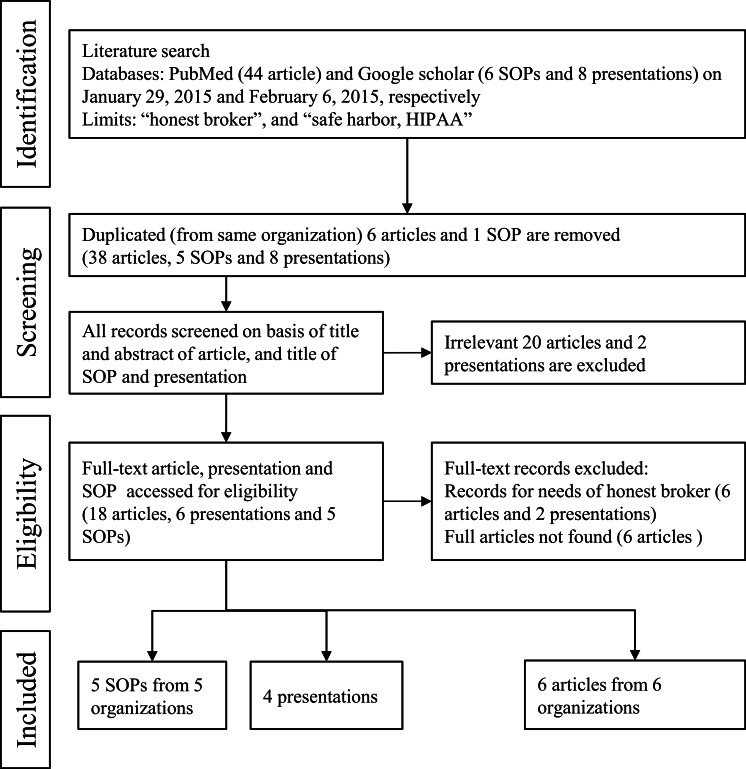
Literature review processes of honest broker in other organizations using PubMed and Google Scholar.

Three researchers (2 honest brokers and 1 medical informaticist) performed a literature review to locate relevant evidence on successful processes or systems that use honest brokers to protect patient private data in clinical research. All literatures were independently reviewed and all data in Table 1 were extracted by the aforementioned researchers. Discrepancies were resolved through discussion. We examined articles from a wide variety of academic fields, including medical informatics, ethics, statistics, and computer science. The 44 articles, 6 SOPs, and 8 presentations identified were evaluated for inclusion in this study. Ultimately, 5 SOPs, 4 presentations, and 6 articles, all of which were originally published as full papers, met the inclusion criteria for this present research (Fig. 1).

### Task force activities for protecting patient privacy data

To prepare for the amended 2012 Korean Bioethics and Safety Act, the AMC developed a Task Force (TF) in 2012–2013 to guarantee legal clinical research. The TF consisted of 15 stakeholders in clinical research (including the departments of biomedical informatics, laboratory medicine, and clinical departments (pulmonology and gastroenterology, and pathology), in addition to the bio-bank, information protection subcommittee, relevant IRBs, and human research protection center). All authors of this paper participated in the TF. The first authors, Ms. Choi and Ms. Lee, who are honest brokers for clinical data, presented the agenda on the necessity, roles, and responsibilities of honest brokers, and with co-author Mr. Lyu, who is working to implement the honest broker system, provided a discussion of the roles and tasks for the system.

The first authors, Ms. Choi and Ms. Lee, who are honest brokers for clinical data, presented the agenda on the necessity, roles, and responsibilities of honest brokers, and the co-author, Mr. Lyu, who is working to implement the honest broker system, provided a discussion of the roles and tasks for the system. Co-authors Dr. Choi, Dr. Lee, and Dr. Shin, who are from the Office of Clinical Research Information at the AMC and who use clinical data as researchers, have accordingly provided counterarguments on the types of data that need to be de-identified for secondary use as well as reasons why this is necessary. Corresponding authors Dr. Park and Dr. Yoo, who are specialists in healthcare informatics and biomedical law and ethics, respectively, have provided a discussion of measures for de-identification of patients’ information, and its legal feasibility.

Since the amended Korean Bioethics and Safety Act requires that (1) any research that uses information that can directly or indirectly identify personal information must receive an IRB review, and (2) consent must be obtained from all research subjects even in the case of retrospective research that does not correspond to the consent waiver condition stated in the Act, the TF conducted research on ways to satisfy the consent waiver conditions while also protecting patient privacy. The following conditions were agreed upon by the TF: (1) establishment of 21 items of institutional personal health information (PHIs) that need to be de-identified when AMC researchers share and use individual patient data for health research purposes; (2) confirmation of the needs, roles, and work scope of the people who act as honest brokers; (3) confirmation of the needs, roles, and work scope of the honest broker system.

## Results

### Review of the literature on honest brokers

Ten organizations were selected from our literature review to evaluate the commonalities and differences in honest broker systems that are currently used in biomedical research (Table 1). Most of these organizations (9 out of 10) built systems to de-identify clinical data automatically. Only one organization (the University of Pittsburgh) maintains 33 certified persons to validate de-identified clinical data. The University of Pittsburgh operates an honest broker certification process that is composed of IRB-mandated education modules, such as Research Integrity, Human Subjects Research in Biomedical Sciences, and HIPAA Researchers Privacy Requirements (Dhir et al., 2008). In particular, the University of Pittsburgh also developed a unique cross-divisional and collaborative broker service for performing multisite clinical research. The basic aim of the honest broker in biomedical research is to de-identify private patient data, and thus almost all organizations target and de-identify the patient PHIs included in clinical data and maintain a master patient index. Of the 10 organizations we reviewed, four target human biomaterials data, such as those included in tissue banks. Most notably, the Duke Comprehensive Cancer Center targets molecular data, such as those gathered by genomic experiments. Interestingly, only six organizations are explicitly compliant with HIPAA privacy regulations, while the other four organizations do not disclose information regarding compliance.

**Table 1:** Summary of honest broker systems in 10 biomedical research organizations.

Organization	Type of honest broker	Type of data	Functions	Compliance	Novel feature
Children’s Hospital of Philadelphia Center for Biomedical Informatics (Children’s Brain Tumor Tissue Consortium, 2015)	System (electronic Honest Broker; eHB)	Clinical and human biomaterial data	Maintains master patient index	No information	Interface functions between multiple systems
Duke Comprehensive Cancer Center (McConnell et al., 2008)	System (Automated Honest Broker Service; AHBS)	Clinical and genomic data	De-identifies patient PHI^a^	No information	System returns the same random key for each unique patient
National Jewish Health (Wade et al., 2014)	System (Integrated Data Repositories; IDR)	Clinical and human biomaterial data	De-identifies patient PHI, maintains master patient index	No information	Customizes the level of de-identification
Ohio State University (Liu et al., 2009)	System (Information Warehouse; IW)	Clinical data	Removes PHI	HIPAA^a^ Privacy regulations	Incorporates with CRDW^a^
University of Arkansas for Medical Sciences Translational Research Institute (University of Arkansas for Medical Sciences, 2015)	System (Honest Broker System)	Clinical, human biomaterial, and image data	De-identifies patient PHI, maintains master patient index	HIPAA Privacy regulations	Interface functions between multiple systems
University of California (Dennis et al., 2006)	System (Central Code Book; CCB)	Patient IDs	Maps patient ID to medical record numbers	No information	No information
University of Chicago Human Imaging Research Office (University of Chicago, 2015)	2 systems (eHB and iBroker)	Clinical and imaging data	Investigator directly records patient record numbers as an anonymized number, maintains a master patient index	HIPAA Privacy regulations	Anonymized image viewer (iBroker)
University of Florida (2015)	System (Honest Broker; HB)	Clinical data	De-identifies patient PHI	HIPAA Privacy regulations	Training course for honest broker
University of Michigan (Boyd et al., 2009)	System (Honest broker; HB)	Clinical data	De-identifies patient PHI, maintains master patient index	HIPAA Privacy regulations	Interface functions between multiple systems
University of Pittsburgh (Dhir et al., 2008)	Several systems (De-ID, Honest Broker System) and 33 certified persons	Clinical and human biomaterial data	De-identifies patient PHI, validates de-identified clinical data	HIPAA Privacy regulations	Honest broker certification process (education module), IRB-approved honest broker service, customizes the level of de-identification

**Notes.**

a PHI, personal health information; HIPAA, Health Insurance Portability and Accountability Act; CRDW, Clinical Research Data Warehouse.

### Institutional definition of PHI

The first step to de-identifying clinical data is to define the list of prohibited identifiers that need to be removed. In the case of HIPAA, the “safe harbor” option can be used as an alternative to full de-identification. Under safe harbor, data are considered de-identified if 17 listed types of identifiers are removed (US Government Publishing Office, 2015a). However, in recognition of the restrictive nature of HIPAA safe harbor practices in clinical research (Clause et al., 2004), HIPAA makes a concession to research organizations by allowing data custodians to release “limited data sets (LDS)” (US Government Publishing Office, 2015b), from which direct identifiers (e.g., name, address, social security numbers, biometric identifiers) are removed but not indirect identifiers (e.g., date of birth, date of death, and treatment dates, which are included on the safe harbor list). The TF at the AMC adopted the safe harbor option from HIPAA, but added new identifiers (resident and foreigner registration numbers and biometric identifiers) as prohibited PHIs in order to comply with domestic laws (Table 2). A more detailed description of the 21 PHIs determined by the AMC are described elsewhere (Shin et al., 2015; Shin et al., 2013).

**Table 2:** The 21 items of personal health information adopted by Asan Medical Center, modified from Table 1 in Shin et al. (2015) (adapted with permission).

No.	Identifier	Remarks	Reference
1	Name	Excludes physician’s name, includes information regarding friends and relatives	HIPAA^a^ safe harbor; HIPAA LDS^a^
2	Address	Smaller than the sub-municipal level divisions (Dong, -Eup, and -Myeon)	HIPAA safe harbor; HIPAA LDS
3	Phone number	Includes mobile phone and fax numbers	HIPAA safe harbor; HIPAA LDS
4	Email address		HIPAA safe harbor; HIPAA LDS
5	Resident registration number		Korean Personal Information Protection Act
6	Foreigner registration number		Korean Personal Information Protection Act
7	Passport number		Korean Personal Information Protection Act
8	Health insurance policy number		HIPAA safe harbor; HIPAA LDS
9	Bank account number		HIPAA safe harbor; HIPAA LDS
10	Credit card number		HIPAA safe harbor
11	Certificate/license number	Driver’s license	Korean Personal Information Protection Act; HIPAA safe harbor; HIPAA LDS
12	Vehicle license plate number		HIPAA safe harbor; HIPAA LDS
13	Patient ID	Medical record numbers	HIPAA safe harbor
14	Hospital membership ID	Hospital homepage, referral system	Korean Act on Promotion of Information and Communication Network Utilization and Information Protection
15	Hospital employee number		HIPAA safe harbor
16	IP address		HIPAA safe harbor; HIPAA LDS
17	URL		HIPAA safe harbor; HIPAA LDS
18	Biometric identifier	Fingerprints, retina, vein, voice prints, and personally identifiable genetic information	HIPAA safe harbor; HIPAA LDS
19	Full-face photographic images and any comparable images		HIPAA safe harbor; HIPAA LDS
20	Birth date (allowing year and month)	e.g., July 1960 can be used, but July 4, 1960 should be used as July **, 1960	HIPAA safe harbor
21	Other unique identifying numbers	Pathology numbers	HIPAA safe harbor

**Notes.**

a HIPAA, Health Insurance Portability and Accountability Act; LDS, Limited Data Set.

### Specified roles of the honest broker system

At the AMC, ABLE (Asan BiomedicaL research Environment) has been used as the honest broker system (anonymized clinical research data warehouse) since March 2014. The role of ABLE is to support research and to allow for searching and viewing of clinical data after the subject identification information has been anonymized. The ABLE includes three main tools: the cohort tool, chart review tool, and extraction tool. First, prior to establishing research plans, the cohort tool identifies cohorts of research participants who correspond to the requested conditions. Using the cohort tool, the researchers only obtain the number of patients within the search conditions. Second, the chart review tool provides more detailed anonymized information. Using the provided research IDs, the user (i.e., researcher) can access de-identified patient charts (including diagnosis, medication, and examination results, prescriptions, radiology reports, operation reports, admission notes, and discharge summaries). Lastly, the “extraction of anonymized clinical data tool” can download the identified results in Excel format. At that time, the downloaded results must link with the corresponding research that approved by the AMC IRB. According to the AMC policy, the ABLE restricts tool access only to our institution’s researchers who are qualified in GCP and ABLE user training. The ABLE has also recorded all data access logs in the above three tools. Based on these logs, honest brokers have monitored investigators’ violations.

### Qualification, Status, and Obligations of honest brokers at Asan Medical Center

The AMC’s first honest broker was introduced in 2005 with the establishment of the Bio-Resource Center (BRC) for managing biomaterials and associated data. The first honest broker in charge of managing clinical data was recruited in May 2010. Following the introduction of the honest broker system and resulting task force for protecting patient privacy data, additional honest brokers were recruited in February 2014.

Honest brokers at the AMC are subdivided into handlers of clinical data or human biomaterials; this is because the characteristics of the two types of de-identifiable data differ, and thus it is advisable for a specialist who is knowledgeable of the characteristics of each type of data to be responsible for de-identification. Subsequently, the requirements for the position are divided into common essential requirements and individual essential requirements. The AMC has concluded that in the case of clinical data, the honest broker must be proficient in IT and capable of conducting validation through the honest broker system (ABLE) with at least 5 years of experience working in a hospital, whereas the honest broker for human biomaterials and associated data would be required to have knowledge of remaining tissues, fluids, and blood, with a license in clinical pathology or a degree in biotechnology.

It is also a mandatory requirement for all AMC honest brokers to complete the Good Clinical Practice (GCP) course, Health Information Privacy and Security (HIPS) course, and IT method for clinical data extraction course before starting their tasks. They are also required to retake these courses every two years for the continuation of their employment. The AMC IRB reviews whether the honest broker has completed the mandatory training courses. In addition, both types of honest brokers must disclose their present and potential conflicts of interest to the AMC IRB before their appointment.

In the case of the honest broker for clinical data, the Office of Clinical Research Information reviews the candidate’s qualifications, after which the President of the AMC appoints the candidate as an honest broker. In the case of the honest broker for biomaterials, the BRC reviews the candidate’s qualifications, after which the President of the AMC makes the appointment. All honest brokers at the AMC function solely as honest brokers, a full-time occupation.

All honest brokers at the AMC have the duty of maintaining the confidentiality of the type of data provided to researchers. This duty comes into effect when an honest broker signs an “Information Protection Confidentiality Agreement.” This Agreement stipulates that the honest broker must take all responsibility for the leakage of any information managed by the honest broker under the AMC SOP and the Korean Personal Information Protection Act. An honest broker who fails to maintain the confidentiality of the data risks being penalized according the SOP and the Act.

### Specified roles of honest brokers at Asan Medical Center

The honest broker for clinical data must perform the following operations when using ABLE: (1) monitor the anonymity verifications of the honest broker system; (2) provide user training; (3) oversee investigator violations such as mismatches between extracted clinical data and IRB-approved research; (4) consult with investigators regarding the method of ABLE use; (5) provide de-identified data when requested (e.g., data collected by government organizations such as the Ministry of Government Administration and Home Affairs, Statistics Korea, Health Insurance & Assessment Services, and National Insurance Services); and (6) report monitoring/inspection results regarding ABLE to the IRB twice yearly.

The honest broker for human biomaterials performs the following functions: (1) provide coded IDs instead of registration numbers for patients who provided biomaterials, and (2) provide de-identified human biomaterials to the corresponding investigators when requested. The honest broker in charge of human biomaterials handles around 250 cases annually. Table 3 summarizes the functions and features of honest brokers and the honest broker system (ABLE).

**Table 3:** Honest broker framework at Asan Medical Center.

Type of honest broker	Type of data	Functions	Compliance	Novel feature
Honest Broker, the System (ABLE^a^)	Clinical and human biomaterials data	De-identifies patient PHI^a^; provides qualified users with de-identified data regardless of time	HIPAA^a^, Korean Personal Information Protection Act	Classifying level of search tool, restricting level of tool only when qualified
Honest Broker (clinical data)	Clinical data	Monitors the verification of ABLE, provides ABLE user training, oversees investigator violations, consults with investigators regarding the method of ABLE use, provides requested de-identified data collected at government organizations to investigators, reports monitoring/inspection results to IRB	HIPAA, Korean Personal Information Protection Act	Expanded roles of human honest brokers for supplementing the honest broker system
Honest Broker (human biomaterials and their data)	Human biomaterials	Provides coded IDs and requested de-identified human biomaterials to investigators	HIPAA, Korean Personal Information Protection Act	Anonymizing human biomaterial itself

**Notes.**

a ABLE, Asan BiomedicaL research Environment; PHI, personal health information; HIPAA, Health Insurance Portability and Accountability Act.

**Figure 2:**
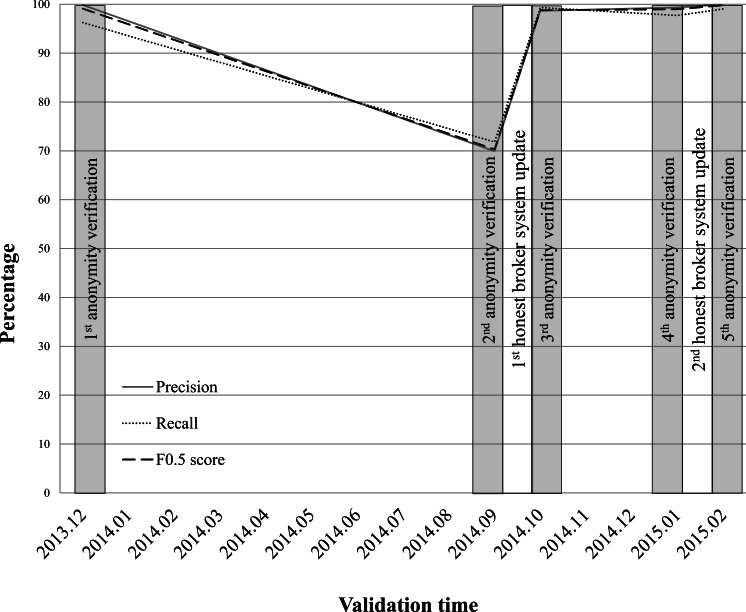
Monitoring results of the anonymity verifications performed by the ABLE system at AMC.

At the AMC, honest brokers have monitored the anonymity verifications of the ABLE system since December 2013. Monitoring of the anonymity verifications of the system by honest brokers should be conducted four times per year. Oversight of investigator violations is performed on 1,800 requests for clinical data extractions per year. Based on the log of the ABLE, the honest broker retrospectively monitors about 650 requests per month. Figure 2 shows the anonymity verification results of the AMC ABLE system. Over 15 months, anonymity verifications were conducted five times.

The performance of the anonymizing function of the ABLE system has dramatically declined since September 2014 (precision = 69.93; recall = 71.81; F0.5 score = 70.3). We have selected 1,275 clinical documents (701, 471, and 103 documents for inpatient, outpatient, and emergency room, respectively) for a second anonymity verification (Table S1). Of 195 identifiers in 1,275 clinical notes, 107 identifiers were correctly masked, 46 non-identifiers were incorrectly masked, and 42 identifiers were not masked. All 88 of the incorrectly identified were found in narrative text in clinical documents, new patterns for describing PHI that are hard to detect by a de-identification algorithm without updating PHI detection rules. After the first honest broker system update, the anonymizing function of the third anonymity verification results (precision = 99.87; recall = 96.25; F0.5 score = 99.12) demonstrated similar accuracy to the first anonymity verification result (precision = 98.66; recall = 99.33; F0.5 score = 98.8). Three months later, the performance of the ABLE system had declined slightly by the fourth anonymity verification (precision = 99.33; recall = 97.7; F0.5 score = 99). After the second honest broker system update, the anonymizing function demonstrated the highest accuracy (precision = 100; recall = 99.05; F0.5 score = 99.81) among all anonymity verifications. A detailed validation data set and the PHIs used for anonymity verification are provided in Table S1.

### Honest brokering processes and publication rules at Asan Medical Center

**Figure 3:**
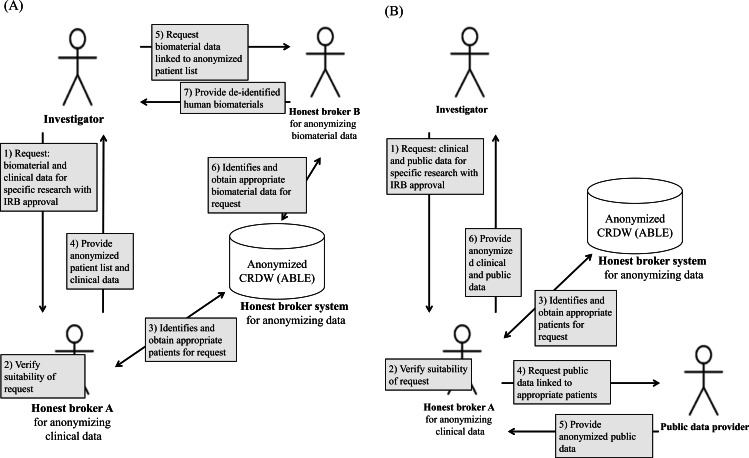
Schematic of the honest broker handling processes at AMC for (A) clinical and human biomaterials data and (B) clinical and public data.

Honest brokering at AMC requires collaboration between humans and systems (Fig. 3). Figure 3A details the honest brokering processes specified by the AMC Honest Broker SOP for both the ABLE system and humans, and for clinical and human biomaterials data. Regardless of the type of study, it is a precondition at the AMC that the investigator obtain AMC IRB approval before conducting a secondary study on existing clinical or human biomaterials data without consent. Accordingly, the AMC IRB must first verify the ethical/legal suitability of the request.

The AMC IRB reviews whether the research protocol of using data for secondary research without the consent of the patient is legal and ethically appropriate. The honest broker provides consultation to the IRB prior to their deliberation as to whether the de-identification of the relevant data is practically or technically feasible, or examines this afterwards. For example, some clinical images are difficult to de-identify due to technical limitations. In this case, even after approval by the IRB, if the request is practically infeasible for de-identification the honest broker reports the results to the corresponding investigator and the IRB.

Figure 3B shows the processing details for data collected by public institutions. If the investigator requests public data (e.g., death or other data about a patient) and clinical data available at the AMC, initially the honest broker for clinical data and the IRB verify the suitability of the request. Second, if the request is appropriate, then the honest broker identifies and obtains the patient lists that meet the requested conditions from the honest broker system. Third, when the honest broker requests public data to public institutions with patient lists, then the public data provider provides anonymized public data that the investigator requested. Finally, the honest broker provides both the anonymized clinical data from the AMC and the public data to the corresponding investigator.

As above, data that has been de-identified for the purpose of conducting studies without the consent of the patient must be guaranteed to remain de-identified when the study results are made public. The researcher receives approval from the AMC IRB on the condition that he complies with the above. Thus, researchers are unable to provide raw data or original data to the public when publishing or presenting the results of the study. This is because the purpose of de-identification is to protect the personal information of the subject whose data is used without consent, whereas the release of raw data to the public is a form of re-identification, which means that the subjects’ confidentiality is not fully guaranteed.

### Establishment of conditions for optimal roles of the honest broker

The roles of honest brokers operating at 10 other representative institutions, including both human and system (computer-based), were analyzed for comparison with the AMC in order to extract optimal roles of honest brokers. Optimal roles were determined for the following functions: (1) sufficient de-identification of clinical data (Boyd et al., 2007); (2) sufficient independence from any form of study team (Rothstein, 2010); (3) thorough examination of secondary use of clinical data for purposes restricted to those approved by the IRB (Benitez & Malin, 2010); and (4) efficient provision of de-identified data to researchers (Patel et al., 2007).

The first condition means that the honest brokers’ role is to be able to sufficiently de-identify PHIs or other clinical identifiers and so ensure regulatory compliance before the release of information to investigators. Furthermore, he or she should monitor the anonymity verification periodically. The second condition means that honest brokers should be an independent party completely separated from researchers, and the only entity able to track the codes linked to clinical identifiers. The third condition means that they should prohibit the use of clinical data for purposes not approved by IRB and report to the IRB in cases when anonymization will not be possible. The fourth condition emphasizes the need for efficient delivery of de-identified data to researchers in a timely and accurate manner.

One condition can be distinct from another, but there is no need for these conditions to be independent from each other. For example, “sufficient de-identification of clinical data” and “efficient provision of de-identified data to researchers” are tasks that can be interconnected. When a researcher wishes to conduct a retrospective study through chart reviews without the consent of a patient, the honest broker de-identifies the data to the extent possible and then delivers this data to the researcher. Hence, the four conditions may affect each other, can be interconnected, and can also facilitate the execution of each other’s roles.

All of these conditions determine the actual success or failure of honest brokers. Here, “sufficient,” “thorough,” or “efficient” does *not* mean “greatest” or “maximum,” but denotes “optimal” practices that honest broker systems need to adopt in order to protect information and promote clinical research with limited resources.

### Evaluations of roles of honest brokers at AMC according to conditions

In the case of the AMC, the introduction of the ABLE somewhat *paradoxically* emphasizes the role of the human component of this system. One of the main functions of the honest brokers is system monitoring for anonymity verification. At the AMC, an enormous amount of anonymity validations were conducted by honest brokers before the ABLE was introduced (Shin et al., 2013). Since most clinical data consist of narrative text, new patterns often emerge. In order to operate a robust and consistent honest broker system, validation strategies need to be established and periodic checkups performed (Fig. 2). At the AMC, after honest broker monitoring, the accuracy of the anonymizing function of ABLE highly increased, although it was still not 100% (i.e., partial satisfaction of condition 1).

In addition, even though the anonymizing function had improved, there still remains an important and unique role for an honest broker in the overall system: that of providing education and consultations to researchers in order to improve the acceptance and use of the ABLE, auditing the authorizations to access the system, and reporting monitoring/auditing results to the IRB (i.e., satisfying condition 3).

The aforementioned roles of the honest broker have wide applicability outside of Korea. There is, however, a role that must be specifically applied to honest brokers in Korea due to various legal requirements. In Korea, government organizations must authorize the release of patient death information. In this case, personal identifiable information collected by research institutions (i.e., the resident registration number (RRN)) must be provided to the corresponding government organization. The honest brokers at the AMC send RRNs to these agencies when there are requests by researchers, who then receive this information from those organizations in a pseudonymized form. In other words, the honest broker at AMC fulfills the role of an “honest” intermediary between government organizations and researchers, such as blocking any possible re-identification by researchers (i.e., satisfying condition 2).

The honest broker frameworks, both system and human, will need to be enhanced in the near future, as reflected by their limitations at AMC and other institutions. In the case of the AMC, the ABLE cannot de-identify some clinical images, such as endoscopic and ultrasonic photographic images that do not comply with DICOM standards (Pianykh, 2009) (i.e., therefore not satisfying condition 1). The variety of honest broker systems, as shown in Table 1 (such as the anonymized image viewer used by the University of Chicago), highlight the need to improve the function of the honest broker process at the AMC. In addition, the University of Pittsburg’s cross-divisional and collective brokering service features an honest broker (both human and system) that is a part of the facility, provides de-identified data to departments/divisions, aggregates and provides specimens and data, and designs software tools for these purposes. The policy that the honest broker system encompasses several separate departments and divisions reduces possible conflicts of interest. Given the fact that fostering an enhanced research output is one of the essential conditions for the honest broker, cooperation between individual honest brokers working in different departments should be actively considered (which is needed to satisfy condition 4).

## Conclusions and Future Research

The roles of the honest brokers discussed herein not only bridge the gap between the idealized concept of the honest broker and its practical needs, but also act as references for other institutions considering ways to bridge gaps in personal health data protections.

While our present study provides reasons for establishing an honest broker process and provides guidance on best practices in this regard, there are several aspects of this issue that require future exploration. First, guidelines are needed that regulate the definitions, purposes, roles, and requirements for honest brokers. There are no currently existing regulations regarding honest brokers, so different institutions autonomously prescribe their own definitions and roles. However, because there are continuing needs for both stronger health data protection and multi-institute/multi-national clinical research, coherent regulations for honest brokers are also needed that promote consistent anonymization of patient data.

Second, agreements between Korean clinical research institutions and national regulatory departments that define the Korean version of LDS would likely be fruitful. In Korea, protecting PHIs is now emphasized by a number of clinical studies, but there is no definitive list of PHIs that should be removed. At the AMC, we adopted part of the HIPAA-defined limited set elements in order to propose a Korean version of the LDS, but this LDS is only applicable to the AMC at present. In order to clearly present the PHIs that all honest brokers must de-identify, an LDS must be established for all Korean institutions and that complies with Korean standards and laws.

Lastly, satisfaction surveys and input of researchers and other stakeholders are needed to improve the quality of honest brokers. Only then will the ultimate goal of the honest brokers—to bridge the gaps between personal health data protection and clinical research efficiency—be achieved.

## Supplemental Information

10.7717/peerj.1506/supp-1 Table S1 Anonymity validation result of honest broker system by honest broker as human Five anonymity validation result using 21 personal health information (PHI) by human honest broker in Asan medical Center. TP, True Positive; FP, False Positive; FN, False Negative.

## Additional Information and Declarations

### Data Availability

The following information was supplied regarding data availability: In our manuscript, clinical documents were used as raw data for validating the anonymity function in our honest broker system. We are not permitted to provide these clinical documents to other organizations.
